# Deep learning for diagnosis of acute promyelocytic leukemia via recognition of genomically imprinted morphologic features

**DOI:** 10.1038/s41698-021-00179-y

**Published:** 2021-05-14

**Authors:** John-William Sidhom, Ingharan J. Siddarthan, Bo-Shiun Lai, Adam Luo, Bryan C. Hambley, Jennifer Bynum, Amy S. Duffield, Michael B. Streiff, Alison R. Moliterno, Philip Imus, Christian B. Gocke, Lukasz P. Gondek, Amy E. DeZern, Alexander S. Baras, Thomas Kickler, Mark J. Levis, Eugene Shenderov

**Affiliations:** 1 Bloomberg Kimmel Institute for Cancer Immunotherapy, Johns Hopkins University School of Medicine, Baltimore, MD USA; 2 The Sidney Kimmel Comprehensive Cancer Center, Johns Hopkins University School of Medicine, Baltimore, MD USA; 3 Department of Biomedical Engineering, Johns Hopkins University School of Medicine, Baltimore, MD USA; 4 Department of Pathology, Johns Hopkins University School of Medicine, Baltimore, MD USA; 5 Division of Hematology, Department of Medicine, The Johns Hopkins University School of Medicine, Baltimore, MD USA; 6 Present Address: Hematopathology Service, Department of Pathology, Memorial Sloan Kettering Cancer Center, New York, NY USA

**Keywords:** Computational biology and bioinformatics, Diagnostic markers, Cancer genetics

## Abstract

Acute promyelocytic leukemia (APL) is a subtype of acute myeloid leukemia (AML), classified by a translocation between chromosomes 15 and 17 [t(15;17)], that is considered a true oncologic emergency though appropriate therapy is considered curative. Therapy is often initiated on clinical suspicion, informed by both clinical presentation as well as direct visualization of the peripheral smear. We hypothesized that genomic imprinting of morphologic features learned by deep learning pattern recognition would have greater discriminatory power and consistency compared to humans, thereby facilitating identification of t(15;17) positive APL. By applying both cell-level and patient-level classification linked to t(15;17) PML/RARA ground-truth, we demonstrate that deep learning is capable of distinguishing APL in both discovery and prospective independent cohort of patients. Furthermore, we extract learned information from the trained network to identify previously undescribed morphological features of APL. The deep learning method we describe herein potentially allows a rapid, explainable, and accurate physician-aid for diagnosing APL at the time of presentation in any resource-poor or -rich medical setting given the universally available peripheral smear.

## Introduction

APL is a subtype of myeloid leukemia that is distinguished clinically by its rapidly progressive and fatal course due to its propensity to cause intracranial bleeding due to fibrinolysis and thrombocytopenia^1–4^. The effective and life-saving treatment of choice for APL is not a chemotherapeutic agent as for other leukemias, but rather all-trans retinoic acid (ATRA) which is an agent that differentiates malignant clonally expanded promyelocytes resulting from the t(15;17) translocation^5–9^. As a true oncologic emergency, obtaining a rapid and accurate diagnosis is of utmost importance in the clinical management of these patients^3,10^. However, definitive widely utilized genetic testing (cytogenetics, fluorescence in situ hybridization (FISH) for t(15;17) or polymerase chain reaction (PCR) for PML/RARA)^11^, can take days to confirm a diagnosis as workflows relating to this rare disease, vary by clinical center, and dictate that tests are often run in batches weekly or sent out externally. Delays of even 48 h to treatment increase morbidity and mortality. Newer confirmatory tests such as reverse transcription-quenching loop-mediated isothermal amplification^12^ and anti-promyelocytic leukemia (anti-PML) antibody^13,14^ have been described, but are not widely used, validated, or incorporated into clinical workflows especially outside of developed countries and tertiary-care centers. Therefore, early treatment decisions are based on clinical suspicion at the time of presentation while additional workup is being pursued.

In an effort to improve diagnostic reasoning, clinicians have utilized the peripheral smear to identify morphological features associated with promyelocytes associated with APL including presence of coarse granules, Auer rods, bilobed nuclei, as well as a low percentage of blasts in the peripheral blood with monocytoid features^15^. Unfortunately, a definitive diagnosis of APL by inspection of the smear can be challenging even for the most experienced hematopathologists. This reality is further compounded by the fact that APL is a rare leukemia with an annual incidence of only 600–800 cases in the United States making prompt recognition especially difficult for providers with limited experience with leukemia^16^. Definitive diagnosis of APL requires molecular confirmation of PML-RARA translocation, but these techniques require time and are not available in many countries with limited healthcare resources. In contrast, the peripheral smear is universally available, and easily and rapidly obtained in all healthcare settings. To this end, we hypothesized that deep learning could be used to differentiate and diagnose APL from other subtypes of myeloid leukemias solely from cellular morphology resulting from genomic imprinting that could be assessed by the power of deep learning pattern recognition^17,18^. In this study, we present a deep learning approach for both descriptive and predictive purposes to learn the morphological features of APL linked to molecular translocation status and leverage this information to rapidly distinguish APL from other forms of myeloid leukemia.

## Results

In order to train and test our models, we collected a total of 106 patients that were seen at The Johns Hopkins Hospital from 2010 to 2020, divided into a discovery and independent prospective validation cohort. Patients were required to have both a peripheral smear and molecular testing by both PCR and/or FISH, collected from the first peripheral smear obtained upon admission, and retrieved from the Johns Hopkins CellaVision database, for the purpose of training and testing the deep learning models (Supplementary Fig. 1 and Supplementary Table 1).

### Single-cell deep learning predicts and reveals morphological features of APL

**Fig. 1:**
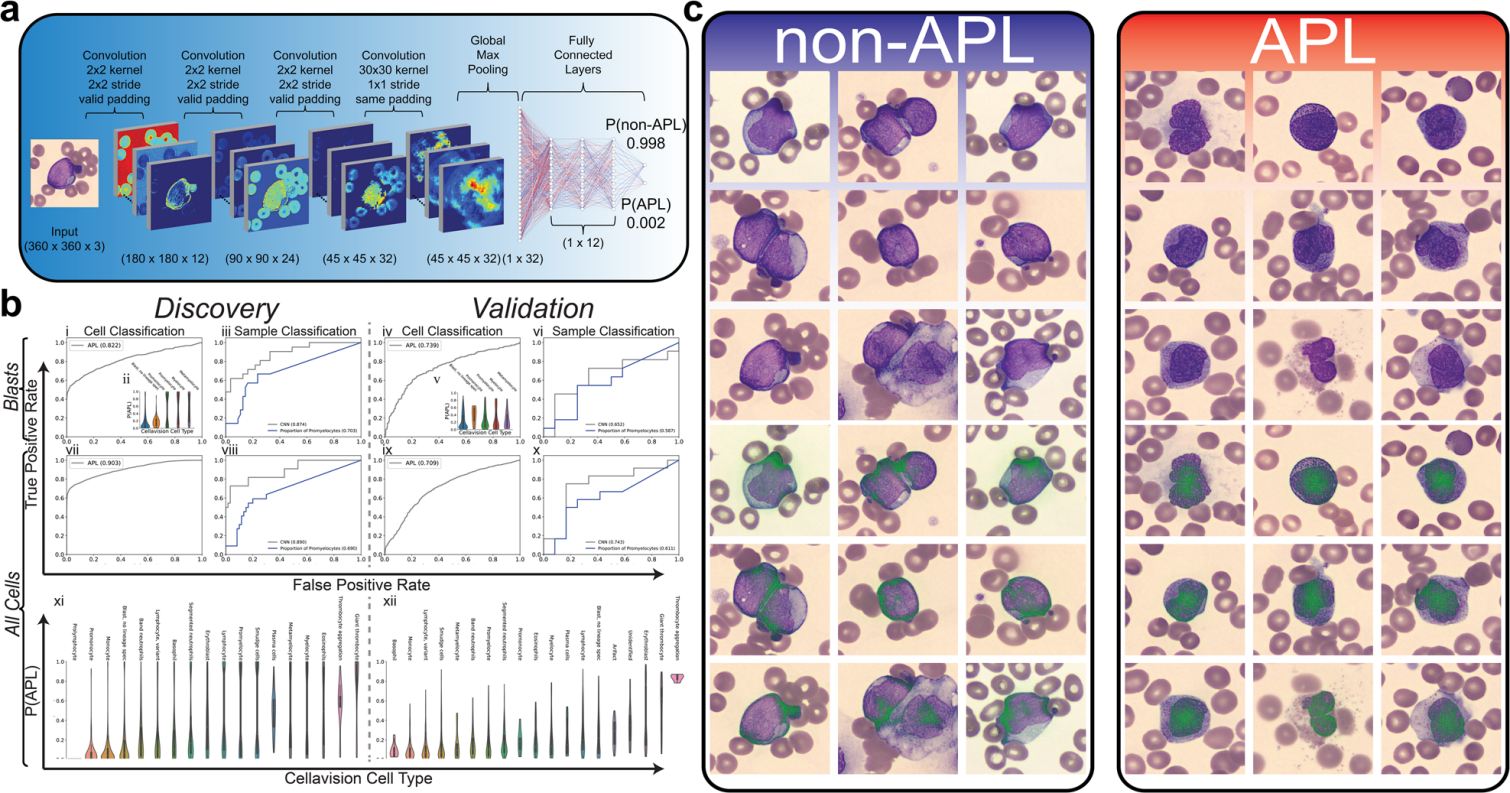
Cell classification from peripheral smears. **a** A deep learning architecture was designed to train cell-level classification of white blood cells taken from peripheral smears. The proposed model takes in each segmented cell from CellaVision and applies four convolutional layers before conducting a global max-pooling operation followed by three fully connected layers and one classification layer to classify each cell as being non-APL/APL. **b** The proposed model was trained where CellaVision data was available on a discovery cohort of 82 patients and tested on an independent prospective validation cohort of 24 patients for which performance metrics are shown. Initially, the model was trained only on immature myeloid cells, here denoted as Blasts, and performance was assessed both at the cell (i,iv) and sample/patient (iii,vi) level for the discovery and validation cohort. Performance was assessed in the discovery cohort in Monte–Carlo (MC) cross-validation and was assessed in the validation cohort by applying the 100 MC models trained in discovery onto the validation cohort in ensemble. Probability of a cell being APL is shown per CellaVision cell type (ii,v). Cell-level predictions were averaged over a given sample to arrive at a per-sample probability of being APL. Sample-level performance was benchmarked against the proportion of promyelocytes within a sample (iii,vi,viii,x). In addition, the model was trained on all cell types from CellaVision, here denoted as All Cells, and performance was assessed both at the cell (vii,ix) and sample/patient (viii,x) level for the discovery and validation cohort. Probability of being APL is shown for all CellaVision cell types in the discovery (xi) and validation (xii) cohorts. **c** After the trained model was applied to the blasts of the validation cohort to assess performance, per-cell predictions were collected and most predictive cells for non-APL/APL were collected and visualized (top row). In addition, integrated gradients were applied to localize the discriminative pixels to provide further information about how the model classified a given cell (bottom row).

We first trained a deep learning model that took input Wright stain images from CellaVision, already incorporated into clinical practice workflows at the Johns Hopkins Cancer Center, and utilized convolutional layers to extract morphological features of the cell for classification (Fig. 1a). We hypothesized that the morphological differences between APL and non-APL leukemias should be present in the immature myeloid compartment and therefore, first applied this model to distinguish APL/non-APL from solely immature myeloid cells (blasts, promonocytes, promyelocytes, myelocytes, metameylocytes). This model had a ROC AUC of 0.822 in the discovery cohort and 0.739 in the validation cohort (Fig. 1b i,iv). Notably, promyelocytes were not the only cell type to carry the predictive signature (Fig. 1b ii,v). To examine the sample classification performance, we took the average per-cell prediction over all cells in an individual as the probability of that individual having APL (Fig. 1b iii,vi). While the performance was high in the discovery cohort, the model did not generalize as well to the validation cohort, even though the model outperformed the proportion of promyelocytes (as a diagnostic biomarker) in both the discovery and validation cohorts. Of note, the proportion of promyelocytes was used as a comparator biomarker for another measurement that could be obtained quickly at the time of admission from the CellaVision algorithm and would be expected to display a monotonic relationship with the probability of a patient having APL given an arrest in cellular differentiation should result in an accumulation of promyelocytes in APL.

In order to reduce our reliance on the CellaVision cell type classifier, we attempted to train a per-cell classifier on all cells regardless of their CellaVision classification (Fig. 1b vii–x). This resulted in improved performance on sample-level classification in the validation cohort when using all cell types even though a model trained on all cells did not generalize as well onto the validation cohort at the per-cell level (Fig. 1b ix,x). When looking at the per-cell predictions by CellaVision cell type, we noted that other cell types carried predictive signatures of APL including platelets (Fig. 1b xi,xii). This finding, while surprising, is consistent with the known propensity of APL to cause bleeding, thrombosis, and DIC. While there are morphological differences in the myeloid compartment between APL and AML, other cell types also provide information useful for distinguishing APL from non-APL leukemia.

Finally, we wanted to understand the morphological differences the neural network had learned in the immature myeloid compartment that distinguished APL from non-APL leukemia. To accomplish this, we used an established method of integrated gradients to identify the distinguishing pixels of the most predictive APL vs non-APL cells (Fig. 1c)^19^. We noted that the AI focused on cytoplasmic pixels in non-APL leukemias and nuclear pixels in APL, possibly consistent with the appearance of the chromatin on Wright staining in the non-APL leukemias being more dispersed and focused at the edge of the cell whereas, in APL, chromatin was more condensed and focused at the center of the cell. These morphological features, taught to us by the model, have not been previously reported in the literature as being useful for distinguishing APL from non-APL.

### Multiple-instance deep learning improves predictive performance

**Fig. 2:**
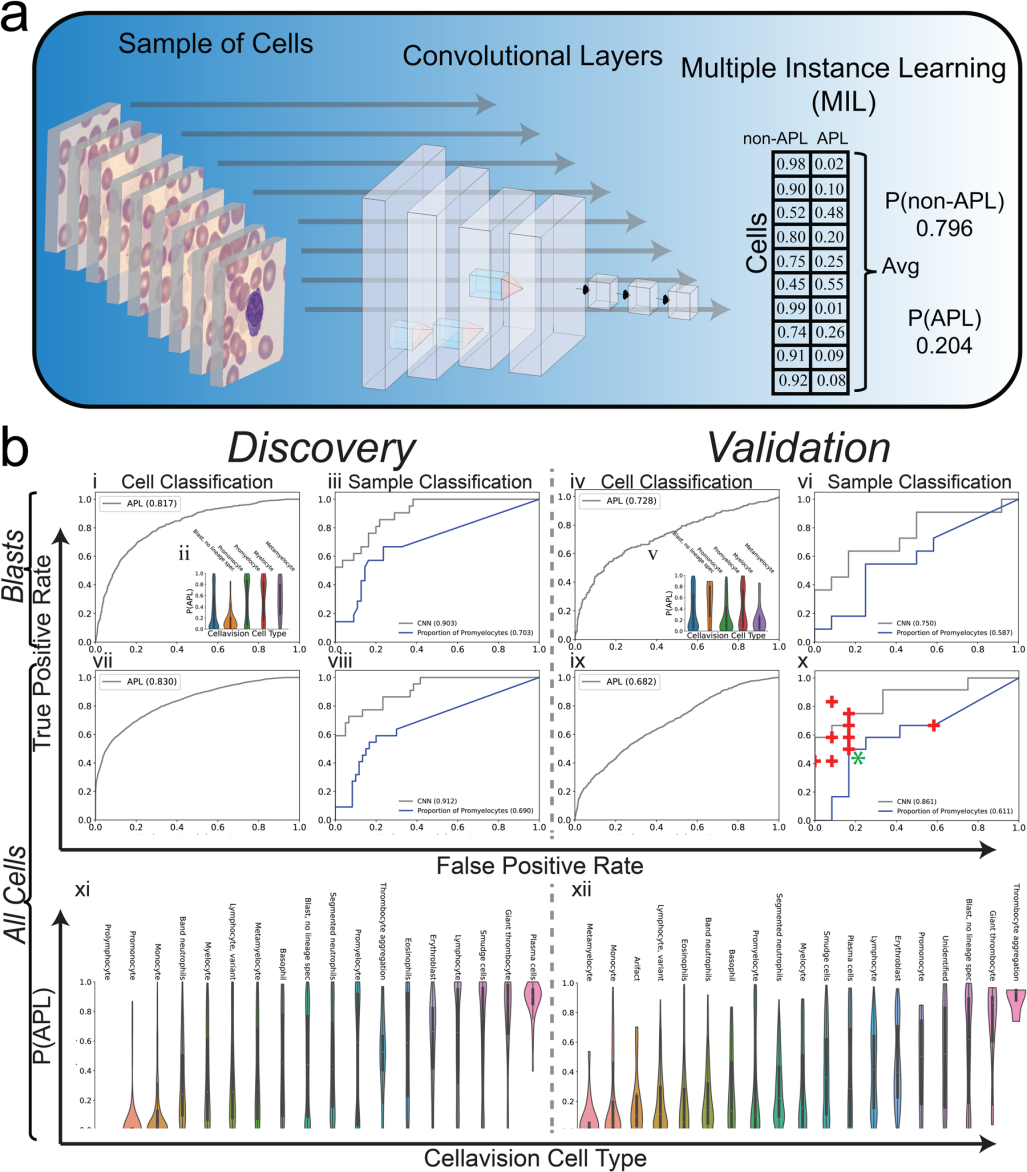
Sample classification via multiple-instance learning approach. **a** A deep learning architecture was designed to train sample-level classification of collections of white blood cells from peripheral smears. The proposed model takes a collection of cells from a given sample/individual and applies the same convolutional and fully connected layers described in Fig. 1 to arrive at per-cell predictions for APL/AML. The per-cell predictions are then averaged over all the cells to arrive at a sample-level prediction. **b** The proposed model was trained where CellaVision data was available on a discovery cohort of 82 patients and tested on an independent prospective validation cohort of 24 patients for which performance metrics are shown. Initially, the model was trained only on immature myeloid cells, here denoted as Blasts, and performance was assessed both at the cell (i,iv) and sample/patient (iii,vi) level for the discovery and validation cohort where cell-level predictions come from cell assignment layer within network and sample/patient predictions come from aggregation layer within the network. Performance was assessed in the discovery cohort in Monte–Carlo (MC) cross-validation and was assessed in the validation cohort by applying the 100 MC models trained in discovery onto the validation cohort in ensemble. Probability of a cell being APL is shown per CellaVision cell type (ii,v). Sample-level performance from the MIL model was benchmarked against the proportion of promyelocytes within a sample (iii,vi,viii,x). In addition, the model was trained on all cell types from CellaVision, here denoted as All Cells, and performance was assessed both at the cell (vii,ix) and sample/patient (viii,x) level for the discovery and validation cohort. CellaVision cells from patients in the validation cohort were provided to 10 clinicians to assess clinician diagnostic specificity/sensitivity against deep learning model (x) (+ denotes an individual clinician. * denotes two individuals with the same performance). Probability of being APL is shown for all CellaVision cell types in the discovery (xi) and validation (xii) cohorts.

While training a cell classifier provided good performance in the discovery cohort, there was a significant decrease in performance in the validation cohort (discovery AUC: 0.890 vs validation AUC: 0.743). We hypothesized that since the label of APL or non-APL applies to the patient, with a known t(15;17) translocation status, and not to any individual cell, applying a multiple-instance learning (MIL) deep learning model would be better suited to solve this problem (Fig. 2a). Our model applies the same convolutional and fully connected layers at the per-cell level as the single-cell classifier and performs a cell-level assignment within the model. However, after this assignment, the average assignment over all the cells is calculated within the network and used to predict whether the collection of cells comes from a patient with APL or non-APL. When comparing the performance of this MIL model with the single-cell classifier, we noted improved performance and better generalization in the validation cohort when using all the cells (Fig. 2b vi,x). Furthermore, when comparing this performance to 10 practicing academic leukemia-treating hematologists, oncologists, and hematopathologists on the validation cohort, the deep learning model demonstrated equivalent or better classification performance (Fig. 2b x).

### Visualization of learned latent space reveals differentiation signature of APL

**Fig. 3:**
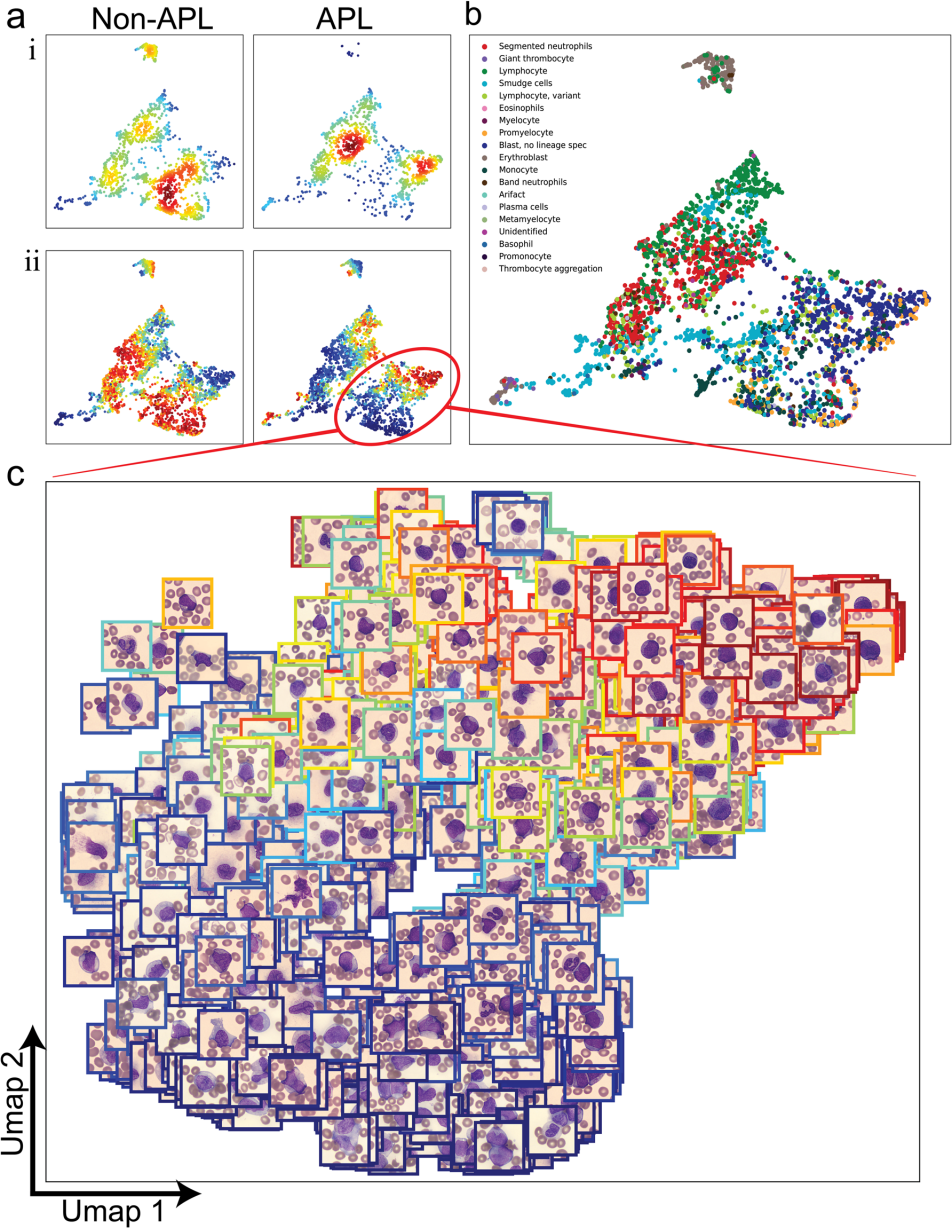
UMAP representation of learned feature space. **a** Per-cell features were obtained by extracting the outputs of the last 4 layers of the trained MIL neural network on all cells in the validation cohort and dimensionality reduction via PCA followed by UMAP was applied to create visualizations. (i) Cells were stratified by non-APL/APL labels and kernel density estimations were calculated to visualize the distribution of cells in this learned feature space where red denotes high-density and blue denotes low-density areas. (ii) Per-cell predictions were extracted from the network and were visualized within UMAP representation (red = high probability, blue = low probability). **b** UMAP labeled by CellaVision cell types. **c** Selected area in UMAP corresponding to immature myeloid cells is highlighted with cell images in respective UMAP coordinates. Color of the surrounding box corresponds to the cell-level probability of being APL (red = high probability of APL, blue = low probability of APL).

To identify what the model had learned, we took the outputs of the MIL model for the last four layers of the network and used these outputs on a per-cell basis within the validation cohort to construct a feature space to visualize these cells in UMAP representation as described in a similar method by Esteva et al.^20^ as well as color them by their probability of being from an APL patient. When visualizing this UMAP representation, stratified by non-APL vs APL (Fig. 3a i,ii), we noted multiple areas of the feature space that were specific for discriminating between non-APL and APL. Furthermore, when analyzed by cell type, we noted that there were differences in the proportion of other cell types that may not have been previously appreciated (i.e., erythroblasts in non-APL, segmented neutrophils in APL) (Fig. 3b). And when looking at the area of the UMAP that corresponded to the immature myeloid compartment, the cellular distribution within this space was consistent with the known biology of finding clonally arrested promyelocytes within APL and more differentiated monocytoid cells within non-APL (Fig. 3c), suggesting that the model had learned the relevant and known biology that distinguishes APL from non-APL.

## Discussion

With future development and prospective validation, the algorithm described herein, already trained on molecular linked imaging data, could be envisioned to serve as a crucial physician-support tool in conjunction with CellaVision or as a cloud application on a smartphone mounted to a microscope for reading peripheral smears in a resource-poor settings that may not have readily accessible molecular diagnostics. Recent work in myelodysplastic syndromes (MDS) showed that using classical morphologic classification criteria, possible pathognomonic relationships between the MDS phenotype and genotype could be identified^21^. In contrast, while this prior work used pathologists to identify the various morphological features associated with the genomic truth, we describe an algorithm using deep learning to learn the morphological features that are predictive of the genotype in acute promyelocytic leukemia.

The current work is limited by lack of external validation using images from outside hospitals (with different scanners) and prospective validation of real-life efficacy in increasing diagnostic performance of clinicians (comparing clinicians plus AI vs. clinicians alone), which will be the focus of future work. Notably, the largest limitation of the work presented is the number of patients we were able to collect data from over the course of this study, explained largely by the low prevalence of APL. Therefore, the findings and conclusion presented within this work should be taken together with this knowledge. In order to best counter this limitation, we made sure to divide our training/validation cohort in time to provide for a prospective validation cohort. However, given the ability of our model to generalize across time within our institution, we hypothesize that as the currently proposed model is able to be trained on data from different institutions, the model would become more robust and generalize more broadly across a variety of clinical sites.

Another limitation of the study was around the number of cells per sample being collected at our institution. For the purpose of our study, only one smear was collected per patient with a maximum of 200 cells per smear being collected via CellaVision. When looking at the performance of our model as a function of the number of cells per patient (Supplementary Fig. 2), we noted that our model was particularly under performing with samples with a low number of cells. Therefore, while a limitation in this current study, a change in future protocol to collect multiple smears per patient would be a low-cost and easily implemented change to improve the robustness of the model.

While the predictive power of the models illustrated within this work is limited by the number of patients within the study, by exploring the interpretability of the learned models, we were able to assess whether the information learned was consistent with already known information about APL. In particular, our models highlighted two key findings including (1) the number of promyelocytes within a given patient was predictive of APL as was appreciated by both using an integrated gradients method to visualize the discriminative features of the most predictive cells as well as visualizing all the cells within a UMAP representation and (2) the presence of other cell types that are associated with known pathology in APL (such as thrombocytes in DIC). The ability to interpret the model and correlate these findings with clinical experience not only provides further confidence in its robustness but provides a method by which a clinician using the tool can understand why the model produced a given prediction, further providing value as a physician-aid in the diagnostic process.

Our work presents deep learning models capable of rapid and accurate diagnosis of APL from universally available peripheral smears. In addition, explainable artificial intelligence is provided for biological insights to facilitate clinical management and reveal morphological concepts previously unappreciated in APL. The deep learning framework we have delineated is applicable to any diagnostic pipeline that can leverage the universally available peripheral blood smear. In the future, we envision a robustly trained and validated algorithm using molecular data linked to morphological features as described herein that can be deployed as a physician-aid in the standard peripheral smear workflow that will allow operator-independent, automated, fully objective scanning of all peripheral smears to alert providers to the probabilistic likelihood of APL, thereby allowing for efficient diagnosis and early treatment of disease in both resource-poor and -rich clinical settings.

## Methods

### Ethics statement

All experiments were conducted in accordance with the Declaration of Helsinki and the International Ethical Guidelines for Biomedical Research Involving Human Subjects. The Human Research Ethics Committee, Johns Hopkins University School of Medicine approved the study.

### Study population

Study patients with APL were identified via retrospective chart review from a list of confirmed FISH t(15;17)-positive patients presenting at The Johns Hopkins Hospital (JHH) who met the inclusion criteria (*n* = 34) of presentation at the time of initial diagnosis, without history of remission, presentation prior to treatment initiation, and availability of peripheral blood smear image uploaded to CellaVision. Other available patient genetic studies, including bone marrow biopsy, cancer karyotype, and PML/RARA mutation status by PCR were examined to confirm the diagnosis for patient selection. Patients were separated into a discovery cohort presenting prior to 1/2019 (*n* = 22) and a validation cohort presenting on or after 1/2019 (*n* = 12).

Patients with AML were identified via retrospective chart review from a list of patients presenting to JHH who at initial presentation had a bone marrow biopsy showing >20% blasts and by acquiring a query of patients who tested negative for the t(15;17) translocation by FISH and who were then confirmed to have AML by bone marrow biopsy and other genetic studies. Those who met the aforementioned inclusion criteria (*n* = 72) were separated into a discovery cohort presenting prior to 1/2019 (*n* = 60) and a validation cohort presenting on or after 1/2019 (*n* = 12). Detailed data set descriptions can be found in Supplementary Fig. 1 and Supplementary Table 1.

### CellaVision

CellaVision™ DM 100 is an automated device for the differential counting of white blood cells (WBCs) and characterization of red blood cells (RBCs). It consists of a slide feeder unit, a microscope with three objectives (×10, ×50, and ×100), a camera and a computer system containing the acquisition and classification software CellaVision™ blood differential software. The number of WBC to be analyzed per patient smear at our institution is set to 200 (user definable from 100 up to 400). To perform a differential count, a thin film of blood is spread on a glass slide from a peripheral blood sample and stained according to the Wright stain protocol. The analyzer performs the acquisition and pre-classification of cells and the operator subsequently verifies and modifies the classification, if necessary.

### Image pre-processing

Data were imported from CellaVision via OpenCV and all images were resized to 360 × 360 pixels via bilinear interpolation for preparation for use of the neural network. Due to a batch effect in the discovery cohort where all individuals collected before 1/1/2018 were individuals with APL, we adopted a novel strategy to deconfound batch effects associated with age of the smear. In particular, the Wright stain used to prepare peripheral smears changes color slightly with age and may exhibit slight variations between preparations, and therefore, devised a method by which to address these batch effects. To do this, we took all training data and applied a gaussian blur, and then used these blurred images as an outgroup for training the model. Therefore, there were three groups of images for training; APL, non-APL, and blurred cells with the goal of having the model learn to distinguish these three classes. The size of the outgroup is equal to the total size of the data set (i.e., the total number of images in the APL and non-APL cohorts). This approach is analogous to conventionally used image augmentation techniques that train a model with augmented images to make them invariant to these features. The difference between using this method over more conventional image augmentation is that we do not need to know, a priori, the features we need to augment to overcome inherent batch effects in the data. By using a blurred outgroup of images, the network is encouraged to learn the differences in the morphology of the cells regardless of confounding features such as those present when applying different staining protocols.

### Deep learning model

The single-cell classifier utilizes four convolutional layers which extract the morphological features of the cells. Following the last convolutional layer, a global max-pooling operation is applied to allow for translational invariance. Following this global max-pooling layer, three fully connected layers + classification layer is used to classify each cell to its appropriate class (APL vs non-APL vs blurred images).

The sample classifier utilizes the same architecture as the single-cell classifier except for the final layers. The model takes in a collection of cells from a given sample. Following per-cell assignment to the output class (i.e., APL vs non-APL vs blurred images), the model takes an average of these assignments (multiple-instance learning) over the entire sample and uses this as the probability of a sample belonging to the appropriate class. All models were built and tested with Google’s TensorFlow™ (v. 1.15.2) deep learning library (https://github.com/tensorflow/tensorflow).

### Training

The single-cell classifier was trained on the discovery cohort in Monte–Carlo cross-validation where 75% of the data was used for training while the other 25% was split in half for validation (to determine convergence) and testing to assess cross-validation performance within the discovery cohort. Notably, test/train was split at the patient/sample level in order to ensure that per-cell performance was tested on individuals the model had not been trained on during cross-validation. This approach would prevent an overestimation of model performance by learning patient-specific features. The single-cell classifier was trained for 100 Monte–Carlo simulations and per-cell predictions were collected only when a given cell fell into the test split. Following training, there are 100 trained models that are used in ensemble on the prospective validation cohort to assess performance in an independent prospective data set. This model was trained for both just immature myeloid cells as well as on all cells regardless of CellaVision cell type.

The sample-level classifier was trained on the discovery cohort in Monte–Carlo cross-validation in the same way as the cell classifier where 75% of the data was used for training while the other 25% was used for assessing cross-validation performance. Due to the small number of samples (in comparison to the number of cells), the model was trained to a certain training loss and then training was stopped. The sample-level classifier was trained for 100 Monte–Carlo simulations in this way and per-sample and per-cell predictions were collected only when a given sample, and its corresponding cells, fell into the test set. The sample-level prediction is collected from the last layer of the model whereas the cell-level prediction is captured by extracting the per-cell assignment from the middle of the network prior to the average aggregation step in the model. In the same way as the cell classifier, the sample classifier was trained for 100 Monte–Carlo simulations and this ensemble of 100 models was applied in ensemble to the prospective independent validation cohort. Another feature of training the sample classifier was the use of sub-sampling each sample per epoch of training where 25 randomly selected cells were chosen for that sample for that particular epoch of training; allowing us to train the model more rapidly while still leveraging all the data over the course of training any individual model. This model was also trained on immature myeloid cells and all cells regardless of CellaVision cell type.

### Integrated gradients

In order to extract interpretability of what the neural network had learned to distinguish APL from non-APL leukemias, we utilized an established method of integrated gradients to localize the predictive pixels for a given input image to its prediction^19^. Our approach takes a given cell and creates 100 scaled images from the blurred version of the image to the original image. These 100 images are sent through a given trained model and the per-pixel gradient is computed between the delta prediction (P(APL)-P(non-APL)) with respect to each pixel to create the attribution map. This was done for 25 pseudo-randomly selected models per image in order to get a consensus of the most important pixels across multiple models for robust and stable attribution maps. Attribution maps were then visualized by overlaying a transparent color scheme over the original image.

### Clinician assessment

De-identified CellaVision images organized by cell type, and representing the whole blood smear as would normally be reviewed in Cellavision for a case, for the 12 APL and 12 AML validation cohort patients were provided to 10 practicing academic leukemia-treating hematologists, oncologists, and hematopathologists who were asked to make a diagnosis solely from the peripheral smear cell images. Their performance was assessed against classifier performance on the validation cohort by determining the true positive rate (TPR) and false-positive rate (FPR) for each of them and plotting this against the ROC Curve for the deep learning model.

### UMAP representations

In order to create visualizations of the learned latent space within the neural network, we extracted the outputs from the last 4 layers of the MIL model for the cells within the validation cohort. These outputs were concatenated together to form a feature space of dimensionality 68. In addition, these 68 features were collected from all 100 models trained in Monte–Carlo in the discovery cohort to total a feature space of dimensionality 6800. Following this per-cell extraction, principal component analysis (PCA) was applied to reduce the dimensionality where enough dimensions were kept to maintain ≥99% of the explained variance. At this point, the UMAP dimensionality reduction technique was applied to bring down the dimensionality to 2 for the purposes of visualization. Kernel density estimation was computed in the UMAP space with the scipy gaussian_kde function. In addition, per-cell predictions for APL were collected for visualization within this UMAP space.

### Statistics

Classifier performance was assessed via area under the receiving operating characteristic (ROC) curve as calculated by scikit-learn (python).

### Reporting summary

Further information on research design is available in the Nature Research Reporting Summary linked to this article.

## Supplementary information

Reporting Summary

Supplementary Figures

Supplementary Data 1

## Data Availability

The image data analyzed during the current study and a metadata table describing the images are openly accessible from the figshare repository 10.6084/m9.figshare.14294675^22^. The imaging data are also available from the Kaggle website https://www.kaggle.com/eugeneshenderov/acute-promyelocytic-leukemia-apl.

## Code availability

All code to reproduce the results from this manuscript as well as all the imaging data can be found at https://github.com/sidhomj/DeepAPL.
