# Supplementary material for: Deep learning for diagnosis of acute promyelocytic leukemia via recognition of genomically imprinted morphologic features
Source: NPJ Precis Oncol. 2021 May 14;5:38. doi: 10.1038/s41698-021-00179-y (PMC8121867; doi:10.1038/s41698-021-00179-y)
Supplement: Supplementary file 1 — Reporting Summary [file 41698_2021_179_MOESM1_ESM.pdf]

nature research

Corresponding author(s): Eugene Shenderov

Last updated by author(s): Feb 6, 2021

Reporting Summary

Nature Research wishes to improve the reproducibility of the work that we publish. This form provides structure for consistency and transparency in reporting. For further information on Nature Research policies, see our Editorial Policies and the Editorial Policy Checklist.

Statistics

For all statistical analyses, confirm that the following items are present in the figure legend, table legend, main text, or Methods section.

n/a	Confirmed	
☐	☒	The exact sample size (n) for each experimental group/condition, given as a discrete number and unit of measurement
☐	☒	A statement on whether measurements were taken from distinct samples or whether the same sample was measured repeatedly
☒	☐	The statistical test(s) used AND whether they are one- or two-sided<br>*Only common tests should be described solely by name; describe more complex techniques in the Methods section.*
☐	☒	A description of all covariates tested
☒	☐	A description of any assumptions or corrections, such as tests of normality and adjustment for multiple comparisons
☐	☒	A full description of the statistical parameters including central tendency (e.g. means) or other basic estimates (e.g. regression coefficient) AND variation (e.g. standard deviation) or associated estimates of uncertainty (e.g. confidence intervals)
☒	☐	For null hypothesis testing, the test statistic (e.g. F, t, r) with confidence intervals, effect sizes, degrees of freedom and P value noted<br>*Give P values as exact values whenever suitable.*
☒	☐	For Bayesian analysis, information on the choice of priors and Markov chain Monte Carlo settings
☒	☐	For hierarchical and complex designs, identification of the appropriate level for tests and full reporting of outcomes
☒	☐	Estimates of effect sizes (e.g. Cohen's d, Pearson's r), indicating how they were calculated

Our web collection on statistics for biologists contains articles on many of the points above.

Software and code

Policy information about availability of computer code

Data collection	All imaging data in this study was collected with the use of CellaVision at our institutution and is available at \url{www.kaggle.com/dataset/3b6d7049abfbf872b22f990b6a4ad6b9a7010ff60927a87ff4fec265fc35e9f4} for public use.
Data analysis	All code to reproduce the results from this manuscript as well as all the imaging data can be found at https://github.com/sidhomj/DeepAPL.

For manuscripts utilizing custom algorithms or software that are central to the research but not yet described in published literature, software must be made available to editors and reviewers. We strongly encourage code deposition in a community repository (e.g. GitHub). See the Nature Research guidelines for submitting code & software for further information.

Data

Policy information about availability of data

All manuscripts must include a data availability statement. This statement should provide the following information, where applicable:

- Accession codes, unique identifiers, or web links for publicly available datasets
- A list of figures that have associated raw data
- A description of any restrictions on data availability

All code to reproduce the results from this manuscript as well as all the imaging data can be found at https://github.com/sidhomj/DeepAPL.

Field-specific reporting

Please select the one below that is the best fit for your research. If you are not sure, read the appropriate sections before making your selection.

☒ Life sciences ☐ Behavioural & social sciences ☐ Ecological, evolutionary & environmental sciences

For a reference copy of the document with all sections, see nature.com/documents/nr-reporting-summary-flat.pdf

Life sciences study design

All studies must disclose on these points even when the disclosure is negative.

Sample size	Study patients with APL were identified via retrospective chart review from a list of confirmed FISH t(15;17)-positive patients presenting at The Johns Hopkins Hospital (JHH) who met the inclusion criteria (n = 34) of presentation at the time of initial diagnosis, without history of remission, presentation prior to treatment initiation, and availability of peripheral blood smear image uploaded to CellaVision. Other available patient genetic studies, including bone marrow biopsy, cancer karyotype, and PML/RARA mutation status by PCR were examined to confirm the diagnosis for patient selection. Patients were separated into a discovery cohort presenting prior to 1/2019 (n = 22) and a validation cohort presenting on or after 1/2019 (n = 12). Patients with AML were identified via retrospective chart review from a list of patients presenting to JHH who at initial presentation had a bone marrow biopsy showing >20% blasts and by acquiring a query of patients who tested negative for the t(15;17) translocation by FISH and who were then confirmed to have AML by bone marrow biopsy and other genetic studies. Those who met the aforementioned inclusion criteria (n = 72) were separated into a discovery cohort presenting prior to 1/2019 (n = 60) and a validation cohort presenting on or after 1/2019 (n = 12).
Data exclusions	N/A
Replication	In order to verify reproducibility of our model, we divided our data collection process into a training set and a prospective independent validation cohort, both at our institution.
Randomization	N/A
Blinding	N/A

Reporting for specific materials, systems and methods

We require information from authors about some types of materials, experimental systems and methods used in many studies. Here, indicate whether each material, system or method listed is relevant to your study. If you are not sure if a list item applies to your research, read the appropriate section before selecting a response.

Materials & experimental systems

n/a	Involved in the study	
☒	☐	Antibodies
☒	☐	Eukaryotic cell lines
☒	☐	Palaeontology and archaeology
☒	☐	Animals and other organisms
☒	☐	Human research participants
☒	☐	Clinical data
☒	☐	Dual use research of concern

Methods

n/a	Involved in the study	
☒	☐	ChIP-seq
☒	☐	Flow cytometry
☒	☐	MRI-based neuroimaging
