# Supplementary material for: Deep learning for diagnosis of acute promyelocytic leukemia via recognition of genomically imprinted morphologic features
Source: NPJ Precis Oncol. 2021 May 14;5:38. doi: 10.1038/s41698-021-00179-y (PMC8121867; doi:10.1038/s41698-021-00179-y)
Supplement: Supplementary file 2 — Supplementary Figures [file 41698_2021_179_MOESM2_ESM.pdf]

# Supplementary Figures

1. **Data set description**
2. **Classification performance as a function of number of cells per sample**

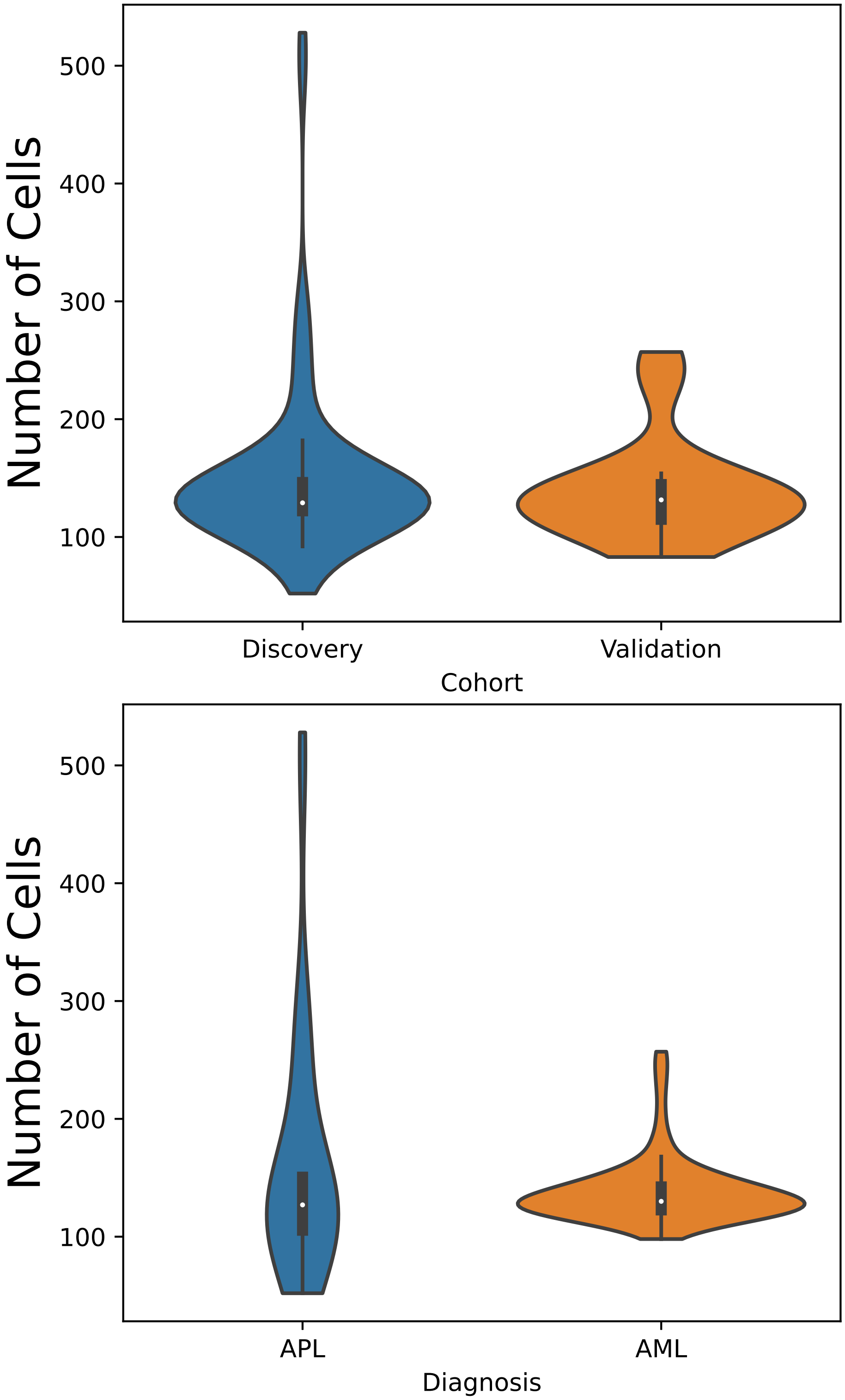


**Supplementary Figure 1. Dataset descriptions** Number of cells per sample was assessed across both discovery/validation cohorts as well as between APL/AML (non-APL) cohorts. Full data can be found in Supplementary Table 1.

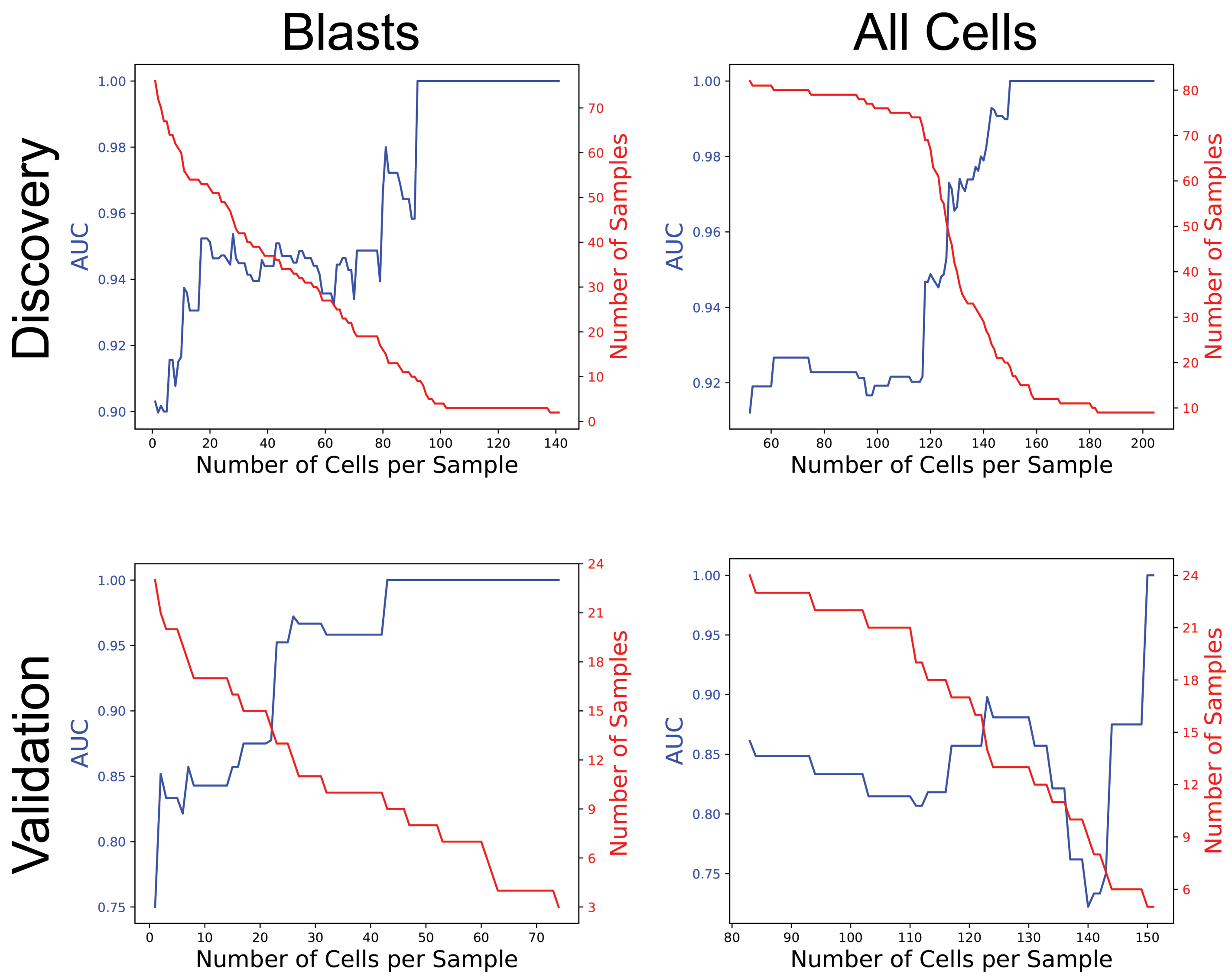


**Supplementary Figure 2. Classification performance as a function of number of cells per sample.** For the multiple-instance learning (MIL) model, ROC AUC was assessed as a function of the number of cells per sample and stratified by both discovery/validation cohorts as well as between blasts/all cells.
